# Effects of *Vibrio parahaemolyticus* on physiology and metabolism of *Thalassiosira weissflogii* in the co-culture system

**DOI:** 10.1128/aem.00323-25

**Published:** 2025-04-17

**Authors:** Jiahui Wang, Mengzhen Cheng, Xin Wang, Guangyuan Wang, Delin Duan, Zhanru Shao

**Affiliations:** 1 College of Life Sciences, Shandong Province Key Laboratory of Applied Mycology, Qingdao Agricultural University 98431, Qingdao, China; 2 State Key Laboratory of Breeding Biotechnology and Sustainable Aquaculture, Institute of Oceanology, Chinese Academy of Sciences 53014 , Qingdao, China; 3 Laboratory for Marine Biology and Biotechnology, Qingdao Marine Science and Technology Center 554912 , Qingdao, China; 4 University of Chinese Academy of Sciences 556346, Beijing, China; Georgia Institute of Technology, Atlanta, Georgia, USA

**Keywords:** chitin, photosynthesis, phycosphere, *Thalassiosira weissflogii*, transcriptome analysis, *Vibrio parahaemolyticus*

## Abstract

Diatoms are crucial primary producers in the marine environment, and their interactions with bacteria exert an important role in the ecosystem. There has been scarce research exploring how diatoms adapt to algal-bacterial environments. In this study, we investigated the physiological and transcriptional distinctions of *Thalassiosira weissflogii* when grown alone (axenic) and with the bacteria *Vibrio parahaemolyticus* (co-culture). Although the bacteria did not significantly impact the growth of *T. weissflogii*, they did affect its photosynthetic efficiency and pigment biosynthesis. The balance of carbon and nitrogen metabolism, as well as energy pathways, including the tricarboxylic acid cycle and glycolysis, was also disrupted. *T. weissflogii* might be capable of maintaining normal growth by upregulating cell cycle-related proteins and utilizing certain bacterial metabolites, such as indole-3-acetic acid. Moreover, *T. weissflogii* reinforced its cell wall in response to *V. parahaemolyticus* infection by increasing chitin biosynthesis and inhibiting chitinase activity. This study explored the effects of *Vibrio* on diatoms from a molecular and metabolic perspective and provided a comprehensive overview of metabolism variations. The results indicate the significant impacts of algal-bacterial interactions on primary producers and offer new insights into the environmental adaptations of diatoms.

**IMPORTANCE:**

The significance of this study lies in its contribution to filling the knowledge gap regarding the interactions between diatoms and pathogenic *Vibrio*. Although extensive research has been conducted on either diatoms or bacteria separately, the mechanisms by which bacteria influence diatom physiological functions and ecosystem processes remain underexplored. Our study reveals that *Vibrio* can significantly alter diatom photosynthesis efficiency and gene expression patterns, providing new insights into how microbial interactions affect element cycling and primary production in marine ecosystems. These findings may have important implications for marine aquaculture, environmental monitoring, and related fields.

## INTRODUCTION

Microalgae represent the most significant primary producers globally. Through the secretion of a wide array of metabolites, microalgae interact with surrounding microorganisms in their environment, creating a unique micro-ecological niche known as the “phycosphere” (1, 2). In this niche, there exists a complex and close interaction between microalgae and bacteria, which is not merely mutualistic or antagonistic, but rather a commensalistic relationship. Bacteria rely on the organic matter produced by microalgae, while microalgae benefit from bacterial production of essential small-molecule nutrients like vitamin B_12_, iron chelators, thiamine derivatives, and phytohormones (3, 4). Some bacteria also secrete algicidal substances to inhibit microalgal growth and vice versa (2, 5). In addition to affecting microalgal physiology through metabolite production, bacteria also regulate their gene expression through multiple signaling pathways. Certain bacteria have been shown to influence gene expression related to photosynthesis and oxidative stress response through the secretion of small-molecule signaling compounds, such as quinones and lipids (6, 7).

Diatoms dominate primary production in marine ecosystems, accounting for approximately 40% of marine primary productivity and 20% of global photosynthetic carbon fixation (8). They are widely distributed across marine, brackish, and freshwater environments, adapting to a range of environmental conditions through the regulation of their physiological processes (9). The growth and metabolic activity of diatoms are profoundly influenced by their surrounding microbial communities, particularly bacteria (10). Recent research highlights how bacterial interactions can regulate diatom physiology and gene expression, affecting their growth, metabolism, and ecological function (11).

*Vibrio* are heterotrophic bacteria widely distributed in marine environments and commonly found in diatom aggregation zones (12). Their abundance in the phycosphere of different algal species may vary considerably due to factors such as environmental conditions, algal species, and growth stages (13, 14). Prior studies, such as those by Tai et al. (15) and Frischkorn et al. (16), have demonstrated that *V. parahaemolyticus* can alter major metabolic pathways and form biofilms when co-cultured with microalgae like *T. weissflogii*, a widely distributed diatom species in coastal ecosystems. Co-culture experiments and metabolomics approaches have further revealed that *Vibrio* can influence the metabolism of diatoms like *Thalassiosira pseudonana* (17, 18).

Given the growing interest in bacteria-diatom interactions, this study focuses on the specific relationship between *V. parahaemolyticus* and *T. weissflogii* to better understand how *Vibrio* affects diatom physiology and gene expression. In this study, we established a two-component system comprising *V. parahaemolyticus* and the diatom *T. weissflogii* to investigate the physiological and transcriptional changes in *T. weissflogii* influenced by *Vibrio*. We found that although *V. parahaemolyticus* had minimal impact on the growth of *T. weissflogii*, it significantly influenced specific metabolic pathways, including the TCA cycle, chitin metabolism, Calvin cycle, glycolysis, gluconeogenesis, and nitrogen metabolism. These altered pathways may reflect an adaptive response of the diatom to the phycosphere, potentially playing a crucial role in the complex and dynamic environments it inhabits. Our study seeks to shed light on these intricate relationships, ultimately advancing knowledge of how bacteria influence diatom metabolism and ecological roles.

## MATERIALS AND METHODS

### Co-culture of bacteria and diatoms

The diatom strain, *T. weissflogii* 9021, was donated by Ningbo University (Ningbo, China) and cultured in an optimized f/2 liquid medium provided by Shanghai Guangyu Biological Technology Co., Ltd. (Shanghai, China) (Table S1) (19). The axenic culture was incubated at 20℃ under a 12 h:12 h light-dark cycle with a light intensity of 150 µmol m^−2^ s^−1^. *V. parahaemolyticus* ATCC 17802 was obtained from the Beijing Microbiological Culture Collection Center (BJMCC) (Beijing, China) and cultured in a 2216E liquid medium (Table S2). Thiosulfate Citrate Bile Salts Sucrose Agar (TCBS) (Table S3) was used for *Vibrio* culturing, with a culture temperature of 30℃.

Cells of *V. parahaemolyticus* and *T. weissflogii* from the seed culture were centrifuged for 10 min at 4,000 rpm, washed twice, and re-suspended in filtered and autoclaved seawater (collected from Qingdao, Shandong, China, 36°4′33.4″N, 120°24′30.7″E). The diatom cell concentration was measured with a hemocytometer (XB-K-25) under a microscope (BX51, Olympus, Tokyo, Japan), and the absorbance of bacterial cells was determined using a PowerWaveHT Microplate Spectrophotometer (BioTek Instruments, Inc., Vermont, USA). Axenic *T. weissflogii* and *V. parahaemolyticus* were inoculated into 1 L of sterilized f/2 liquid medium at a cell number ratio of 1:25 for the co-culture experiment. The conditions of the control group were the same as those described above but adding bacteria. The final concentration of diatom cells was 3 × 10^4^ cells/mL, and the final concentration of bacteria was 7.5 × 10^5^ CFU/mL. After 30 days of co-culturing, the medium was replenished with fresh medium for semi-continuous culture. The cells were collected by centrifugation, rapidly frozen in liquid nitrogen, and stored at −80℃ for subsequent experiments.

### Determination of growth and photosynthetic parameters

The cell density of *T. weissflogii* was determined using a microscopic counting method. The algal suspension was counted using a hemocytometer every 72 h. Each sample was measured in triplicate, and the average value was calculated to evaluate the effects of *V. parahaemolyticus* on the growth of *T. weissflogii*.

Chlorophyll content was determined using the method by Zhu et al. (20). A 50 mL algal sample was centrifuged at 5,000 rpm at 4℃ for 15 min. The pellet was frozen in liquid nitrogen for 30 s, then thawed at room temperature for 5 min, with this process repeated four times. After the cell disruption treatment, 5 mL of 90% acetone was added to resuspend the pellet mixture, and the tube was placed in the refrigerator at 4℃ for 4 h. The sample was then centrifuged at 8,000 rpm for 15 min. Finally, absorbance was measured at 630 nm and 664 nm wavelengths, and Eq. (1) was used to calculate chlorophyll content (21).


(1) $$\begin{array}{rl} & \text{ chlorophyll a }(\mu g/mL)=11.47\times A_{664}-0.40\times A_{630}\\ & \text{ chlorophyll }c_{1}+c_{2}\;(\mu g/mL)=24.36\times A_{630}-3.73\times A_{664} \end{array}$$

where A664 and A630 represent the absorbance at 664 nm and 630 nm, respectively. Chlorophyll *a* and Chlorophyll *c* represent the content of chlorophyll *a* and chlorophyll *c*, respectively.

Variations in chlorophyll fluorescence parameters provide indirect insights into photosynthetic activity (22). In this study, chlorophyll fluorescence parameters were measured using a FluorCam chlorophyll fluorescence imaging system (Photon Systems Instruments, Brno, Czech Republic). Briefly, 5 mL of algal samples was taken and kept in the dark for 15 min to ensure the complete shutdown of all PSII reaction centers. Then, the efficiency of light energy conversion (*Fv/Fm*) within the PSII reaction center was recorded.

### Determination of carbon and nitrogen content

The samples, snap-frozen in liquid nitrogen, were freeze-dried under vacuum at −55℃ for 48 h. Carbon (C) and nitrogen (N) contents were quantified using a Vario EL III elemental analyser (Elementar Analysensysteme GmbH, Frankfurt, Germany) with the following operating parameters: Furnace 1: 950℃; Furnace 2: 500℃; Furnace 3: 0℃.

### Determination of chitinase activity

Approximately 0.05 g of frozen algal cells was taken and resuspended in 1 mL PBS. The sample was sonicated for 6 min (3 s of sonication followed by 10 s of pause), then centrifuged at 10,000 rpm at 4℃ for 10 minutes to collect the supernatant. The Chitinase Activity Kit (Beijing Solarbio Science and Technology, BC0820) was used for the enzyme activity assay.

### RNA extraction and sequencing library construction

Total RNA was isolated using Trizol Reagent (Invitrogen Life Technologies, California, USA). The concentration and purity were determined using a NanoDrop spectrophotometer (Thermo Scientific, Waltham, Massachusetts, USA). Integrity was assessed using RNA-specific agarose gel electrophoresis and the Agilent 2100 bioanalyzer (Agilent Technologies Inc., California, USA). An amount of 3 μg of RNA was used as input material for RNA sample preparation. Sequencing libraries were generated following the steps outlined below.

Eukaryotic mRNA contains a polyA tail, which allows for specific binding with oligo(dT) to selectively enrich eukaryotic mRNA from total RNA. In this study, mRNA with a polyA structure was enriched using Oligo (dT) magnetic beads, and the mRNA was then randomly fragmented by divalent cations. cDNA was synthesized using fragmented mRNA as the template and random oligonucleotides as primers. The double-stranded cDNA was purified, amplified by PCR, and the library was then obtained. The library size was assessed using the Agilent 2100 Bioanalyzer, and the total and effective concentrations of the library were quantified by real-time quantitative PCR (RT-qPCR).

After RNA extraction, purification, and library construction, the libraries were subjected to paired-end sequencing by Shanghai Personalbio Biotechnology Co., Ltd. (Shanghai, China) using the Illumina NovaSeq 6000 sequencing platform.

### Transcriptome analysis

For data quality control, we used fastp (v0.22.0) software to filter the sequencing data and obtain high-quality sequences (Clean Data) for further analysis (23). The filtered reads were mapped to the reference genome using HISAT2 (v2.1.0) (https://daehwankimlab.github.io/hisat2/). HTSeq (v0.9.1) software was used to screen differentially expressed genes (DEGs), and principal component analysis (PCA) was performed to analyze gene expression differences. The read count for each gene was calculated as its original expression, and Fragments Per Kilo bases Per Million fragments (FPKM) was used to standardize expression levels. Differential gene expression was analyzed using DESeq2 (v1.38.3) with the following screening criteria: |log_2_FoldChange| > 1 and a significant *P*-value < 0.05 (24). GO enrichment analysis was performed using topGO (v2.50.0), and the *P*-value was calculated using the hypergeometric distribution method (with a significance threshold of *P*-value < 0.05) to identify significantly enriched GO terms for differential genes, thus determining their main biological functions (25). KEGG pathway enrichment analysis was performed using clusterProfiler (v4.6.0) software (26), with a focus on significantly enriched pathways with a *P*-value < 0.05.

### RT-qPCR validation

To verify the reliability of the RNA sequencing expression results, we selected six key genes for qPCR analysis. The 2× SYBR Green qPCR Mix was purchased from SparkJade (AH0101-A, SparkJade Biotechnology Co., Ltd., Shandong, China). The primers used for qPCR are listed in the supplementary data (Table S4). The reference gene used for qPCR was actin (27). Relative expression quantification was calculated using the 2^–ΔΔCt^ method (28).

### Statistical analysis

In this study, all experiments were performed in triplicate, and the means ± standard deviation (SD) were calculated. All biochemical and physiological data were plotted using GraphPad Prism 8, and independent samples t-tests were performed using SPSS version 26 (*P* < 0.05). In addition, transcriptome data were analyzed on the Personalbio Gene Cloud platform.

## RESULTS

### Growth and photosynthesis

**Fig 1:**
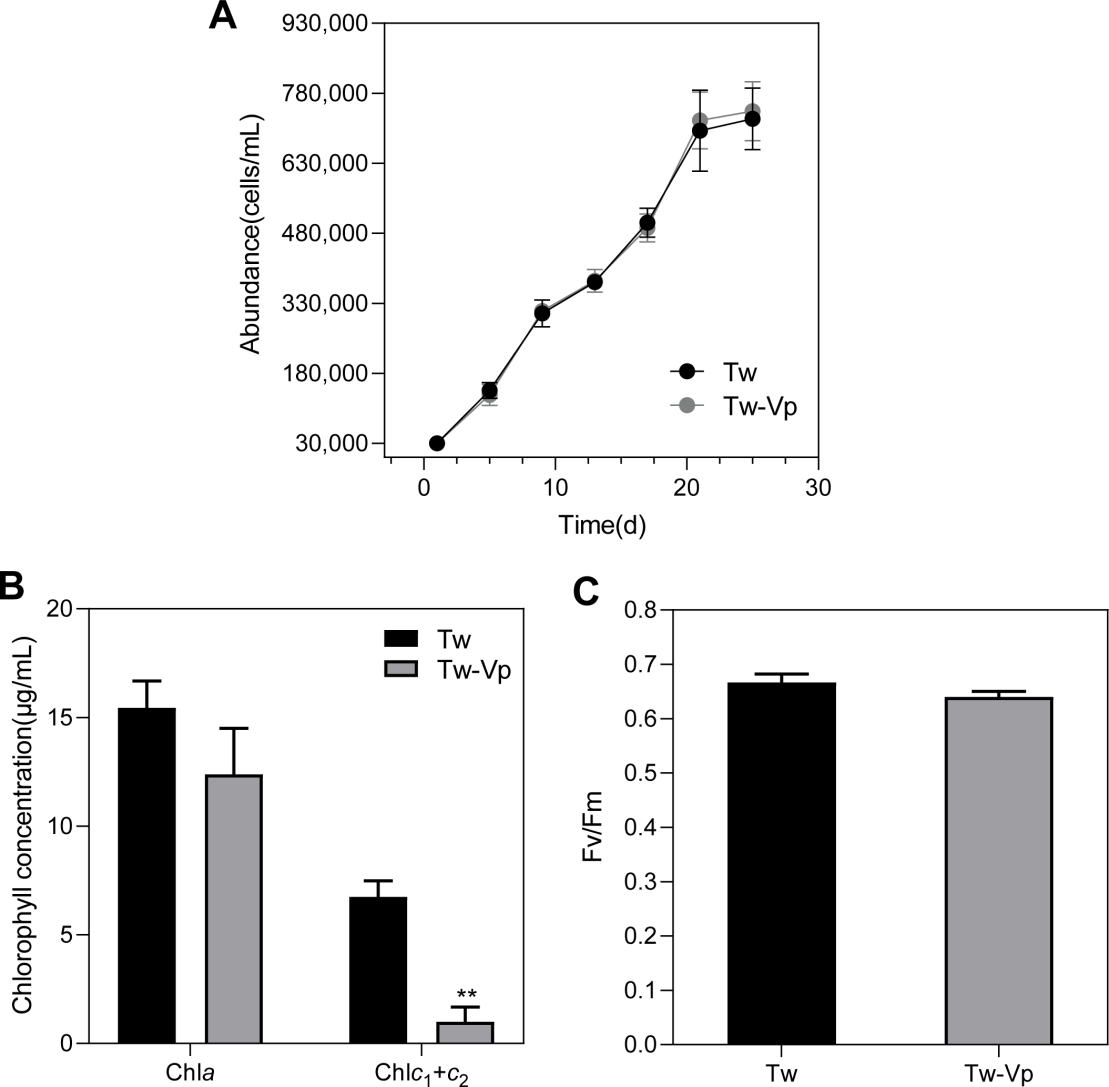
The impact of *V. parahaemolyticus* on the physiological indices of *T. weissflogii*. (**A**) The growth curves of the 30-day co-culture. (**B**) Chlorophyll content. (**C**) The maximum photosynthetic efficiency (*Fv/Fm*) of PSII. The statistical significance is indicated by * *P* < 0.05, **P < 0.01. The data were analyzed using a Student’s t-test with a sample size of *N* = 3. The bars represent the mean values ± SD. The abbreviations Tw and Tw-Vp refer to the strains of *T. weissflogii* and *T. weissflogii* co-cultured with *V. parahaemolyticus*, respectively.

The growth of axenic and co-cultured *T. weissflogii* was nearly identical, indicating a negligible effect of *V. parahaemolyticus* on the growth of *T. weissflogii*. After 20 days, the growth rate of both groups decreased significantly (Fig. 1A). The contents of chlorophyll *a* and chlorophyll *c* were used as indirect indicators of *V. parahaemolyticus*-induced alterations in photosynthesis in *T. weissflogii*. The results showed no significant change in the chlorophyll *a* content of *T. weissflogii* in the presence of *V. parahaemolyticus*, while the chlorophyll *c* content exhibited a significant reduction of 85.25% compared to the control group (*P* < 0.01) (Fig. 1B). Furthermore, the maximum quantum efficiency of PSII (*Fv/Fm*) did not significantly change under co-culture conditions compared to axenic culture conditions (Fig. 1C).

### Chitinase activity and C:N ratio

**Fig 2:**
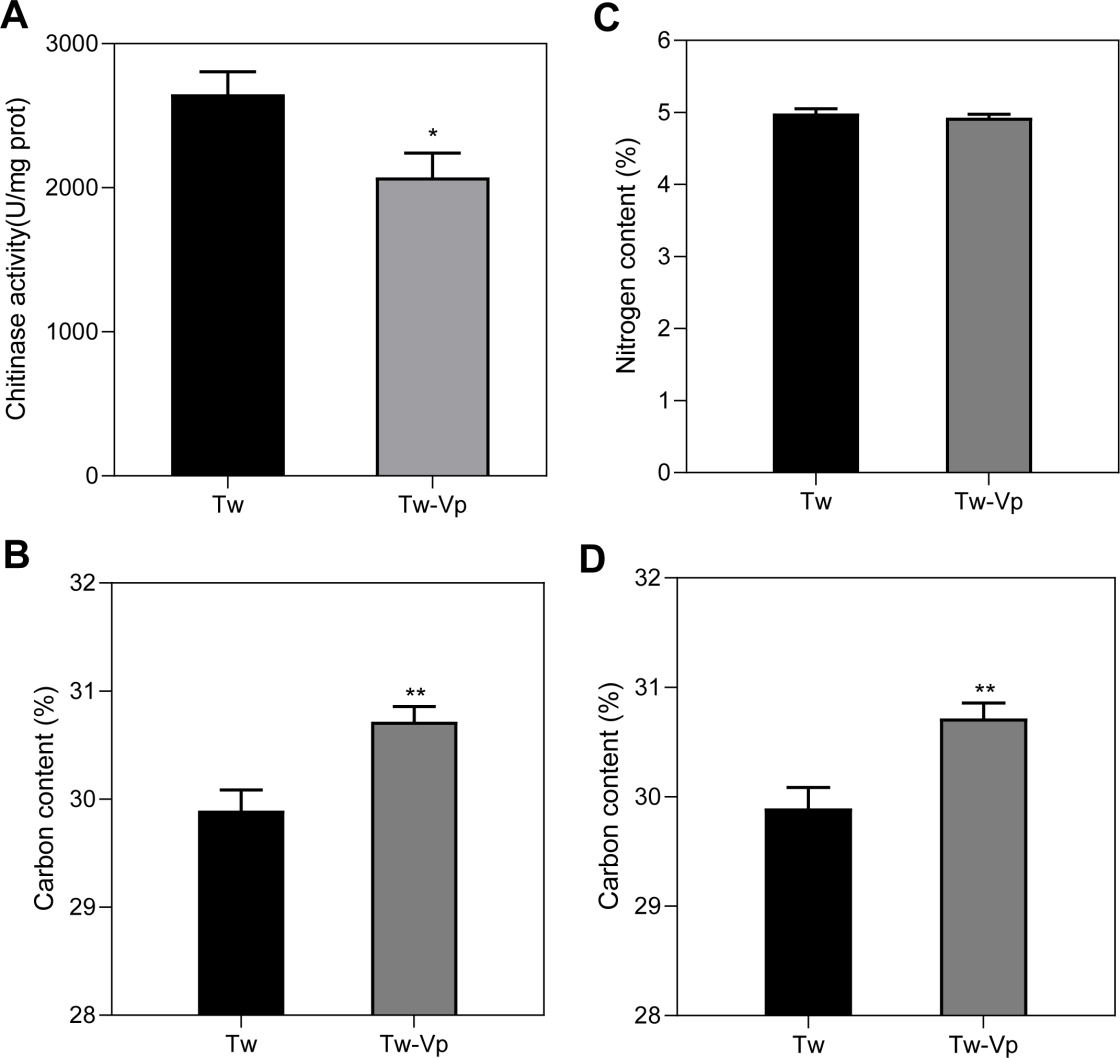
The impact of *V. parahaemolyticus* on the physiological indices of *T. weissflogii*. (**A**) Chitinase activity. (**B**) Carbon content. (**C**) Nitrogen content. (**D**) C:N ratio. *P < 0.05, **P < 0.01. *N* = 3. Student’s t-test. Bars = means ± SD.

Chitin, the most abundant amino polysaccharide in the cell wall of *T. weissflogii*, can be degraded by chitinase into oligosaccharides. Chitinase activity in the co-culture was 21.8% lower than that in the control (*P* < 0.05) (Fig. 2A), indicating a significant downregulation of chitinase. Analysis of carbon and nitrogen, the two basic elements of *T. weissflogii*, revealed that the carbon content in the experimental group was 2.75% higher than in the control group (*P* < 0.01), accounting for approximately 30.72% of the dry weight (Fig. 2B). There was no significant change in the nitrogen content, which accounted for approximately 4.9% of the dry weight (Fig. 2C). In addition, the C:N ratio increased by 4.63% in the experimental group (*P* < 0.01) (Fig. 2D).

### Quality analysis of RNA-Seq data

Six cDNA libraries were constructed, and high-throughput sequencing generated an average of 7,007.8 Mb of raw data and 46.4 Mb of original reads (Table 1). After filtering out low-quality reads and splices, the mean length of clean data and clean reads for each sample was 6,894.7 Mb and 45.8 Mb, respectively. Comparison of filtered reads with the reference genome yielded an average mapping rate of 81.39% and a GC content of 48.08%. The mean Q20 and Q30 values were 98.36% and 95.38%, respectively, indicating a high degree of base identification accuracy and minimal contamination during sequencing. Principal component analysis (PCA) was performed using gene expression values to assess the similarity between sample groups. The results revealed significant differences between the two groups, with high reproducibility (Fig. S1). The contribution values of PC1, PC2, and PC3 were 93.2%, 3.3%, and 1.8%, respectively.

**TABLE 1:** Transcriptome data of *T. weissflogii* under the influence of *V. parahaemolyticus*

	Maximum	Minimum	Average
Raw read no.^*a*^	51,023,908	40,637,742	46,409,018
Raw data (bp)^*b*^	7,704,610,108	6,136,299,042	7,007,761,668
Clean read no.^*c*^	50,427,866	39,999,262	45,754,957
Clean data (bp)^*d*^	7,601,508,117	6,026,179,727	6,894,708,751
Mapped read no.^*e*^	32,580,520	41,021,406	37,821,833
Mapping ratio^*f*^	81.65%	81.25%	81.39%
GC (%)^*g*^	48.31%	47.73%	48.08%
Q20 (%)^*h*^	98.43%	98.27%	98.36%
Q30 (%)^*i*^	95.48%	95.15%	95.38%

^*a*^ Raw read no.: total number of raw reads.

^*b*^ Raw data (bp): total number of bases.

^*c*^ Clean read no.: number of reads of high-quality sequences.

^*d*^ Clean data (bp): number of bases of high-quality sequences.

^*e*^ Mapped read no.: total number of sequences compared to the reference genome.

^*f*^ Mapping ratio: the percentage of total mapped/clean reads.

^*g*^ GC (%): GC content.

^*h*^ Q20 (%): percentage of bases with base identification accuracy above 99%.

^*i*^ Q30 (%): percentage of bases with base identification accuracy above 99.9%.

### Analytical identification, clustering, and functional enrichment of DEGs

**Fig 3:**
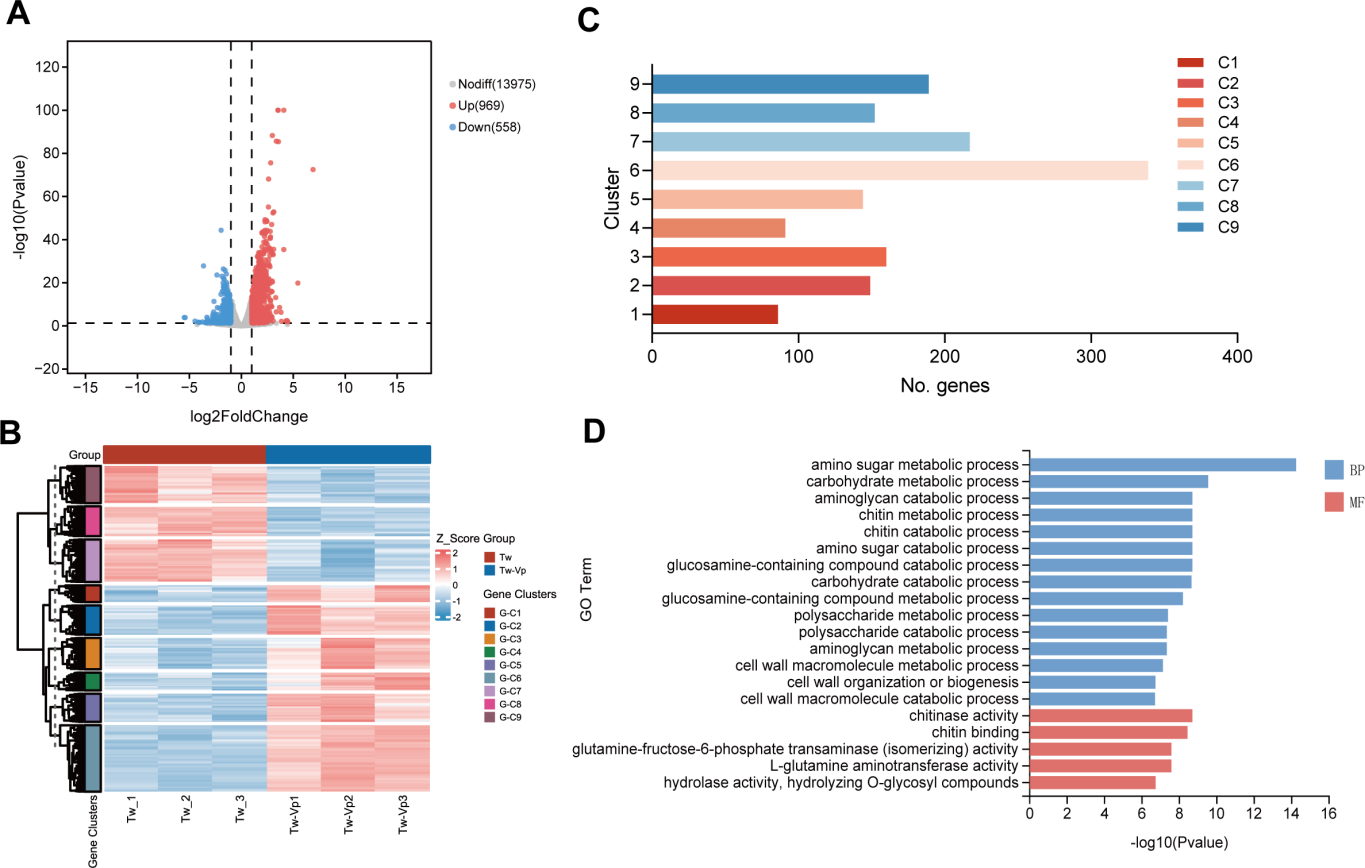
Transcriptome analysis. (**A**) DEGs volcano plot between pure culture and co-culture. The two vertical dashed lines in the plot are the thresholds for expressing fold change, and the horizontal dashed line is the significance level threshold. Red indicates that the gene is upregulated, blue indicates that the gene is downregulated, and gray indicates non-significant differential expression. (**B**) Supplementary description of the gene clustering heatmap, representing the number of genes within each cluster. (**C**) Cluster analysis heatmap for DEGs. Group: different colors represent distinct groups. Gene clusters: genes with similar expression patterns are clustered into a cluster. (**D**) GO enrichment analysis of DEGs in co-cultured *T. weissflogii*, blue represents biological processes while red represents molecular functions.

A total of 1,536 DEGs were identified between the experimental group and the control group, with 969 upregulated and 558 downregulated (Fig. 3A). To investigate gene expression patterns under co-culture conditions, a bi-clustering analysis of DEGs was conducted (Fig. 3B). The results showed similar gene expression trends within each group, indicating a high degree of correlation in gene expression. However, significant differences in gene expression patterns were observed between the two groups, suggesting substantial alterations following the co-culture of *V. parahaemolyticus* and *T. weissflogii* (Fig. 3B). According to the bi-clustering heat map analysis, genes were divided into nine distinct clusters based on the similarity of their expression patterns. These clusters were classified into two categories: clusters 1–6 showed an upward trend in gene expression, while clusters 7–9 showed a downward trend (Fig. 3C; Fig. S2).

To functionally annotate transcriptional changes, KEGG enrichment analysis of the genes in these clusters was conducted (Table S5). The results indicated that DEGs were primarily involved in metabolic pathways and environmental adaptation. Specifically, an upward trend was observed in amino sugar and nucleotide sugar metabolism, as well as plant-pathogen interaction, while a downward trend was noted in photosynthesis-antenna protein and porphyrin metabolism.

The molecular changes in *T. weissflogii* in response to *V. parahaemolyticus* were investigated by analyzing DEGs using GO enrichment to identify their main biological functions (Fig. 3D). In the classification of biological processes, amino polysaccharide metabolism, chitin metabolism, and cell wall component metabolic processes appeared multiple times. We traced the gene clusters under each term and found that chitinase genes played a significant role in these biological processes (Fig. 3D). In the classification of molecular functions, DEGs were primarily associated with chitinase activity, chitin binding, glutamine-fructose-6-phosphate transaminase (isomerizing) activity, L-glutamine aminotransferase activity, hydrolase activity, and the hydrolysis of O-glycosyl compounds (Fig. 3D). Both biological processes and molecular functions were related to polysaccharide metabolism, particularly the chitin metabolism pathway, suggesting that this process is significantly influenced by *V. parahaemolyticus*.

### *V. parahaemolyticus*-induced stress-responsive genes

To gain insights into the mechanisms by which *T. weissflogii* maintains homeostasis in the presence of *V. parahaemolyticus*, we performed an in-depth analysis of genes that may be involved in the defense process in diatoms (Table 2; Table S6). The results showed a significant upregulation of cyclin-encoding genes, which are essential for cell proliferation. In addition, we observed a downregulation of the gene encoding amidase, which catalyzes the production of indole-3-acetamide, a precursor of IAA. We hypothesize that the reduction in indoleacetic acid biosynthesis in *T. weissflogii* could be attributed to the presence of *V. parahaemolyticus*. Diatoms possess intrinsic defense mechanisms, and we identified two upregulated genes encoding antimicrobial peptides (AMPs), which can inhibit the growth of *V. parahaemolyticus*. In addition, differential expression of oxidative stress-related genes was observed, including upregulation of catalase and peroxidase, and downregulation of glutathione reductase, glutathione S-transferase, catalase, and L-ascorbate peroxidase. Notably, genes encoding calcium-dependent protein kinases (CDPKs) were significantly upregulated, suggesting a potential enhancement of signal transduction pathways in *T. weissflogii* (Table 2; Table S6).

**TABLE 2:** Genes differentially expressed between axenic and co-cultured *T. weissflogii* groups

Classification	Gene ID	log_2_FC	*P*-value	Annotation
Porphyrin metabolism	mikado.scaffold_11G157	−1.43	1.86 × 10^−3^	Porphobilinogen synthase
	mikado.scaffold_7G437	−3.74	1.43 × 10^−2^	Uroporphyrinogen decarboxylase
	mikado.scaffold_6G863	−1.56	1.75 × 10^−26^	Coproporphyrinogen III oxidase
	mikado.scaffold_6G865	−1.04	5.20 × 10^−4^	Coproporphyrinogen III oxidase
Photosynthesis	mikado.scaffold_25G214	−2.40	4.85 × 10^−2^	Fucoxanthin-chlorophyll *a-c* binding protein
	mikado.scaffold_20G145	−2.35	2.45 × 10^−24^	Fucoxanthin-chlorophyll *a-c* binding protein
	mikado.scaffold_19G274	−1.06	2.41 × 10^−9^	Ferredoxin
	mikado.scaffold_19G368	−1.71	1.17 × 10^−7^	Ferredoxin
	mikado.scaffold_29G23	1.70	4.54 × 10^−16^	Cytochrome c554
	mikado.scaffold_32G105	2.34	2.87 × 10^−4^	Cytochrome c554
Signal transduction	mikado.scaffold_7G634	2.96	1.29 × 10^−16^	Calcium-dependent protein kinase
	mikado.scaffold_12G352	2.67	5.06 × 10^−37^	Calcium-dependent protein kinase
Cell cycle	mikado.scaffold_8G645	2.12	8.64 × 10^−21^	G1/S-specific cyclin-D1
	mikado.scaffold_6G207	1.77	1.76 × 10^−3^	G2/mitotic-specific cyclin-1
	mikado.scaffold_7G324	1.17	1.56 × 10^−7^	Cyclin-A1-2
	mikado.scaffold_30G84	1.72	3.54 × 10^−33^	Cyclin-D6-1
Antioxidase	mikado.scaffold_3G555	6.90	3.26 × 10^−73^	Catalase
	mikado.scaffold_10G33	1.16	8.49 × 10^−15^	Cytochrome c peroxidase
	mikado.scaffold_9G492	1.09	4.65 × 10^−4^	Glutathione peroxidase
	mikado.scaffold_14G519	−2.13	4.76 × 10^−2^	Glutathione S-transferase
	mikado.scaffold_12G193	−1.13	9.27 × 10^−9^	L-ascorbate peroxidase
	mikado.scaffold_20G53	−1.07	2.89 × 10^−3^	Glutathione reductase

### Expression dynamics of metabolic pathways in *T. weissflogii* during co-culture

**Fig 4:**
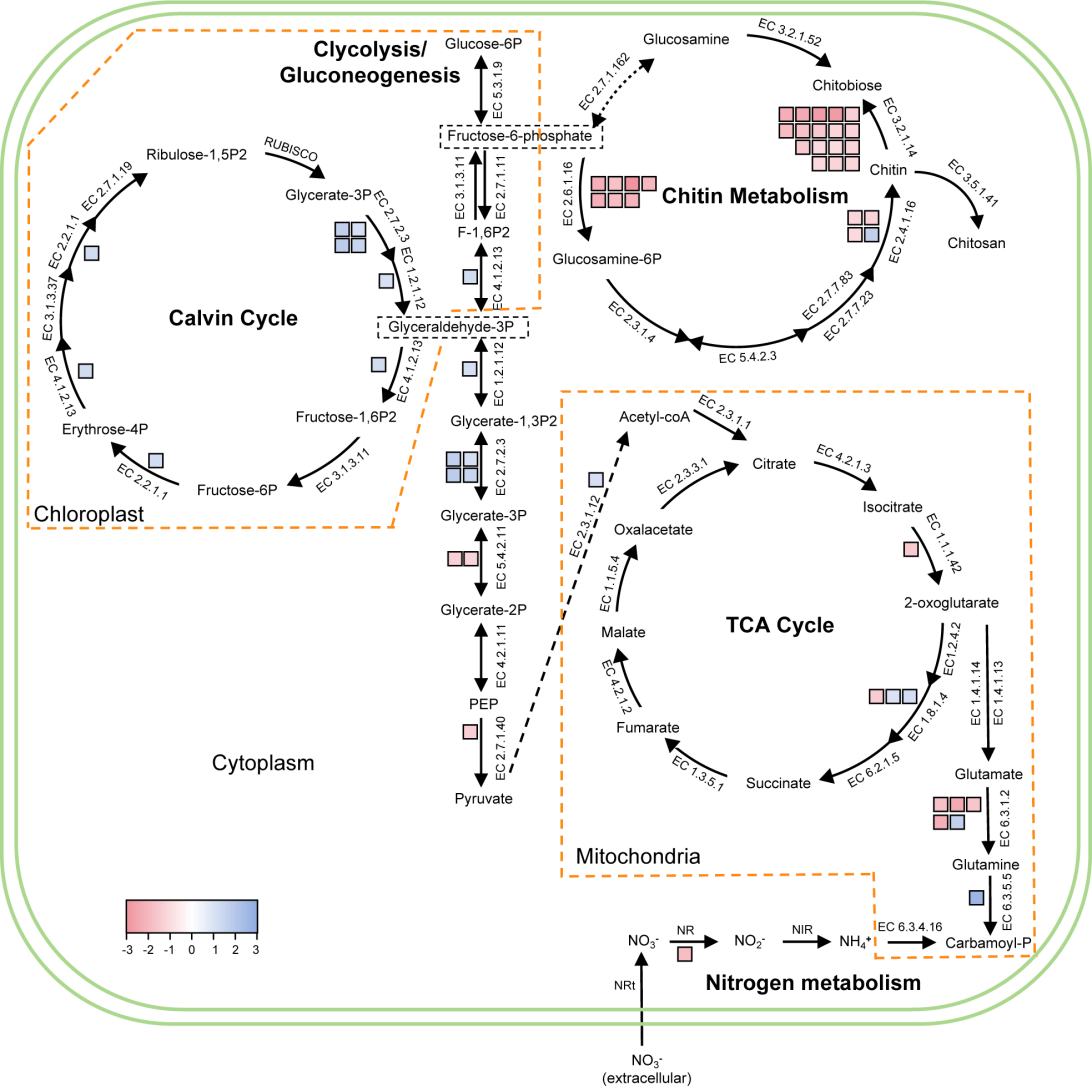
Expression dynamics of metabolic pathways in *T. weissflogii* during co-culture. Each colored box represents a DEG, with red indicating upregulation and blue indicating downregulation. Enzymes encoded by multiple paralogous homologous genes are represented by multiple boxes. Dashed lines define different organelles. The framework reference was Fig. 5 from Downey et al. (29).

We used the KEGG database to enrich DEGs and explore changes in the pathways of *T. weissflogii* under the influence of *V. parahaemolyticus*. Figure 4 summarizes the effects on several crucial pathways.

**Fig 5:**
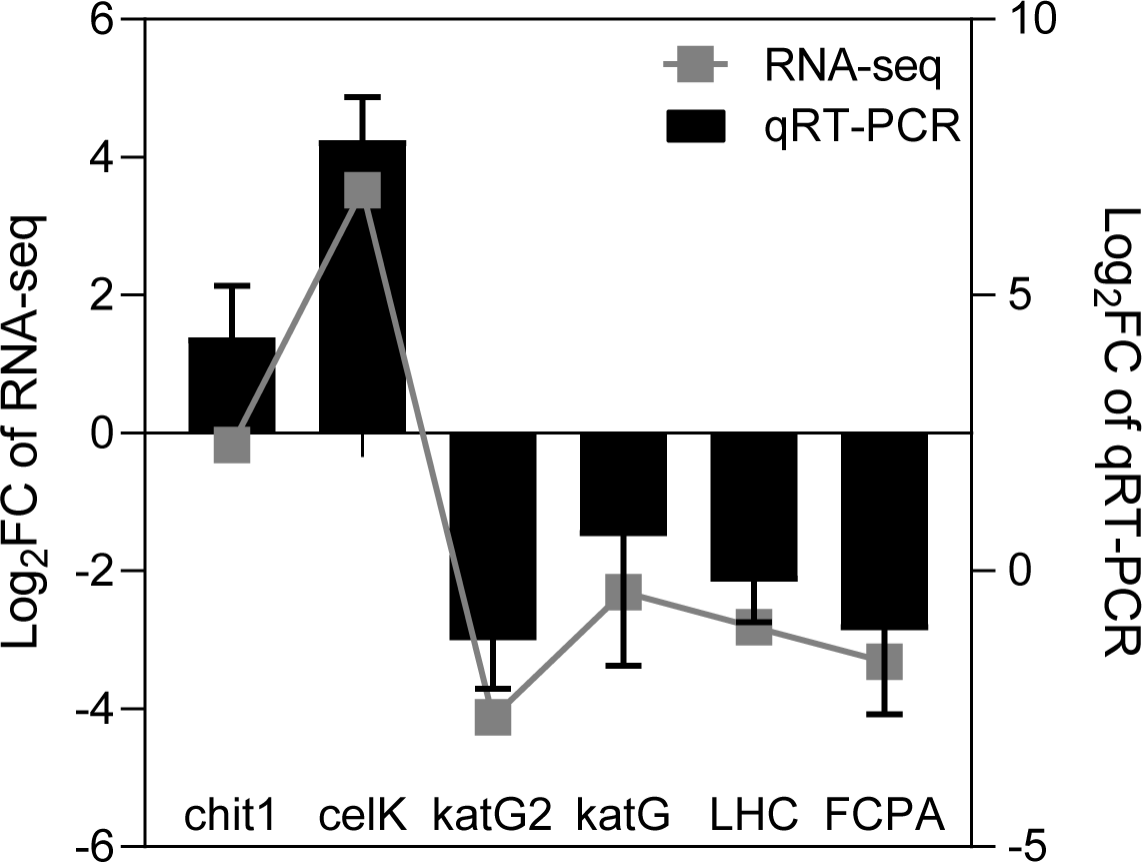
Expression levels of the genes involved in *T. weissflogii* by RNA-Seq and real-time qPCR validation. The housekeeping gene “actin” was selected as an internal reference gene. Values are expressed as the mean of three replicates (± SD) for each treatment. The histogram shows the results of RT-qPCR, and the line graph shows the results of RNA-Seq.

#### TCA cycle

The gene encoding isocitrate dehydrogenase, which serves as a regulatory enzyme in the citric acid cycle, showed a slight increase in expression during the co-culture period (log_2_FC = 1.50). Dihydrolipoamide dehydrogenase (E3) is one of the components of the alpha-ketoglutarate dehydrogenase complex. In the co-culture, one gene was upregulated (log_2_FC = 1.41), while two were downregulated (log_2_FC = −1.00/–1.16). Both enzymes are key players in the TCA cycle, catalyzing two oxidative decarboxylation reactions.

#### Photosynthesis and carbon fixation

The genes involved in chlorophyll synthesis were predominantly downregulated, including those encoding porphobilinogen synthase (log_2_FC = −1.43), uroporphyrinogen decarboxylase (log_2_FC = −3.74), and coproporphyrinogen III oxidase (log_2_FC = -1.56/–1.04). In addition, 10 out of the 43 genes encoding the diatom-specific light-harvesting protein (Fucoxanthin chlorophyll *a*/*c*-binding protein, FCP) were significantly downregulated (mean log_2_FC = −1.55). The remaining genes showed no change. Similarly, four enzymes (transketolase (log_2_FC = −1.19), fructose-bisphosphate aldolase (log_2_FC = −1.23), glyceraldehyde 3-phosphate dehydrogenase (log_2_FC = −1.07), and phosphoglycerate kinase (mean log_2_FC = −1.51)) in the Calvin cycle were also downregulated, while the two genes encoding Rubisco showed no differential expression. Meanwhile, genes encoding photosynthesis-related proteins were downregulated, such as photosystem II oxygen-evolving enhancer protein 3 (log_2_FC = −1.08) and ferredoxin (log_2_FC = −1.71/–1.06). By contrast, nine genes encoding Cytochrome c554 were upregulated (mean log_2_FC = 1.45), suggesting enhanced intracellular electron transport.

#### Glycolysis and gluconeogenesis

To gain deeper insight into the metabolic alterations in glycolysis and gluconeogenesis, this study focused on rate-limiting enzymes and irreversible steps. In glycolysis, the conversion of phosphoenolpyruvate (PEP) to pyruvate represents an irreversible step, and the upregulation of genes encoding pyruvate kinase (PK) enhances pyruvate production (log_2_FC = 1.37). Pyruvate can be converted to oxaloacetate (OAA), thereby participating in gluconeogenesis. In gluconeogenesis, the gene encoding the rate-limiting enzyme fructose-1,6-bisphosphatase (FBPase) remained unaltered. However, the gene encoding fructose-bisphosphate aldolase (FBA) exhibited a consistent expression pattern in both gluconeogenesis and the Calvin cycle.

#### Chitin metabolism

GO enrichment analysis identified several chitin-related terms. Concurrently, the KEGG database was also enriched with the chitin metabolic pathway. Therefore, we hypothesize that chitin biosynthesis may be associated with cellular defense mechanisms. The gene encoding glutamine-fructose-6-phosphate transaminase (GFPT), the initial enzyme in the chitin biosynthesis pathway, exhibited a significant increase in expression during co-culture (mean log_2_FC = 2.16). It initiates this process by breaking down the fructose-6-phosphate (F6P) molecule into glucosamine 6-phosphate. Notably, the genes encoding chitinase were significantly upregulated (mean log_2_FC = 1.61), while the genes encoding chitin synthase exhibited three upregulated genes (mean log_2_FC = 1.15) and one downregulated gene (log_2_FC = −1.65), indicating active chitin metabolism involved in the response to *V. parahaemolyticus*.

#### Nitrogen metabolism

The interconversion between glutamate and glutamine plays a crucial role in nitrogen assimilation, transport, and cycling (30). Four genes encoding glutamine synthetase (GS) were upregulated (mean log_2_FC = 2.00), while one was downregulated (log2FC = −1.65). In addition, one gene encoding carbamoyl-phosphate synthase (CPS) was downregulated (log_2_FC = −2.64). These findings suggest an increase in GS activity, while catabolism is reduced. The increase in glutamine provides additional nitrogen for the synthesis of other biomolecules, including amino acids, nucleic acids, and proteins. The gene encoding nitrate reductase (NR) was upregulated (log_2_FC = 1.85), indicating enhanced nitrogen assimilation.

### Real-time qPCR validation

We selected six genes (chit1, celK, katG2, katG, LHC, and FCPA) from the transcriptomes for qPCR validation. These genes are involved in photosynthesis, antioxidant activity, and chitin metabolism. Our analysis showed that the expression trends of the six genes in both the RNA-seq and qPCR results were consistent, indicating the reliability of the high-throughput transcriptomic sequencing(Fig. 5).

## DISCUSSION

*V. parahaemolyticus* is a common pathogenic bacterium found in marine environments. It adheres to the chitinous fibers of *T. weissflogii* through the use of type IV pili (16). However, the molecular mechanisms and consequences of *V. parahaemolyticus* interactions with diatoms remain to be elucidated. There is a growing recognition of the pivotal role of microalgal-bacterial interactions in shaping the structure and function of the phycosphere (2). This study investigated the physiological and genetic changes in *T. weissflogii* under co-culture conditions with *V. parahaemolyticus*. The results demonstrated that the growth rate and overall health of *T. weissflogii* remained largely unaltered. However, the balance of carbon and nitrogen metabolism was disrupted, and the process of photosynthesis may be altered. In addition, transcriptome sequencing revealed that the Calvin cycle, glycolysis, TCA cycle, nitrogen metabolism, and chitin metabolism were affected by the co-culture of *T. weissflogii* and *V. parahaemolyticus*.

### No significant changes in the growth of *T. weissflogii*

The presence of *V. parahaemolyticus* does not exert a discernible effect on the growth of *T. weissflogii* (Fig. 1A). This observation suggests that the interaction between the diatom and *Vibrio* may not be simplistically characterized as either growth promotion or inhibition. A dynamic equilibrium may exist when diatoms are subjected to bacterial infestation. Landa et al. (31) reported that the presence of bacteria did not affect the maximum abundance of phytoplankton compared to sterile controls. However, the resting stage was prolonged for both species under bacterial presence. Similarly, Ferrer-González et al. (32) found no evidence of altered diatom growth rates in the presence of bacteria. Furthermore, Olofsson et al. (33) found that a 2-week co-culture with bacteria did not affect diatom growth. These studies support our findings regarding the growth of *T. weissflogii*. In this study, significant upregulation of genes encoding cell cycle proteins was observed through transcriptome sequencing, including G1/S-specific cyclin-D1, G2/mitotic-specific cyclin-1, cyclin-D6-1, and others (Table S7). These proteins regulate various phases of the diatom cell cycle by forming complexes with cell cycle-dependent kinases (CDKs) (34). The proliferation of diatoms largely depends on ordered cell division, regulated by these proteins (35). To adapt to changes in the external environment, algae may adjust their cell cycle progression by upregulating the expression of cell cycle proteins, thereby ensuring regular division and proliferation (36, 37). Furthermore, specific cell cycle proteins may enhance resilience to pathogens by modulating rapid cell division or apoptosis in plant cells. It has been demonstrated that when plants are subjected to pathogen infection, cell cycle regulation can alter cell wall thickness and division patterns, thereby enhancing disease resistance (38). In this study, the expression of genes encoding cell cycle proteins was upregulated when *T. weissflogii* was co-cultured with *Vibrio*, which may help diatom cells maintain a relatively stable growth rate and enhance their pathogen resistance.

It is important to note that our results also revealed a reduction in the biosynthesis of indoleacetic acid (IAA) in *T. weissflogii* (Table S7). IAA is a plant growth hormone that not only regulates plant growth but also plays a crucial role in plant-microbe interactions. It has been demonstrated that *V. parahaemolyticus* can also secrete IAA (39), leading us to postulate that the reduction in IAA synthesis in *T. weissflogii* may be associated with its provision by *V. parahaemolyticus*. It has been proposed that algae and bacteria may act in concert to regulate IAA biosynthesis and secretion, maintaining a dynamic equilibrium of IAA levels that supports algae growth. This equilibrium has been confirmed in studies of plant root-associated microorganisms (40). Since photosynthesis and carbon fixation are somewhat affected in *T. weissflogii*, as discussed below, these alterations in periodic proteins and growth hormones may have been made to balance these processes, thereby explaining the lack of significant changes in growth observed. However, the IAA hypothesis requires further verification through the investigation of physiological variations in *Vibrio* under co-culture conditions.

### Alteration of photosynthesis and carbon fixation in *T. weissflogii*

The *Fv*/*Fm* ratio demonstrates that *T. weissflogii* remained photosynthetically active despite the downregulation of some photosynthesis-related genes. We speculate that other pathways or genes related to photosynthesis might compensate for the downregulated genes, allowing photosynthetic activity to remain stable. A reduction in chlorophyll *a* and chlorophyll *c* content was observed, accompanied by a decrease in FCP expression levels (Fig. 1B). FCP is an efficient light-trapping complex composed of fucoxanthin and chlorophyll, crucial for diatoms to adapt to low-light conditions in aquatic environments (41, 42). Furthermore, the Calvin cycle was affected, with multiple enzymes showing downregulation, indicating a reduced capacity for carbon fixation (Fig. 4). The evolutionary development of photorespiration represents a metabolic pathway that evolved over time to adapt to environmental changes and enhance stress resilience (43). Peterhansel et al. (44) demonstrated that photorespiration may change photosynthesis and carbon fixation. This may explain the observed changes in photosynthesis and carbon fixation. In addition, Shi et al. (45) demonstrated that photorespiration plays a role in the salt stress response mediated by CrHPR1, uncovering a function beyond its conventional metabolic processes.

### Changes in carbon and nitrogen metabolism of *T. weissflogii*

Nutrient exchange plays a crucial role in microalgae-bacteria interactions, involving macronutrients such as carbon and nitrogen, as well as trace elements like vitamins (3, 46). Figure 4 shows that central carbon metabolism, including glycolysis and the TCA cycle, was affected by *Vibrio* co-incubation. Paul et al. (17) found that metabolites exuded by bacteria typically stimulate the metabolism of *T. pseudonana*, with higher concentrations of amino acids, fatty acids, and certain C_4_ sugars under co-culture conditions. Bartolek et al. (10) demonstrated that both intracellular and extracellular carbohydrates increased in *T. pseudonana* cultures following bacterial exudate treatment. Nitrogen is a key component of proteins, nucleic acids, and certain amino acids, playing a crucial role in plant growth and development. An illustrative example is the mutualism between legumes and rhizobia, where rhizobia fix nitrogen and make it available to legumes (47). Similarly, a relationship exists between nitrogen-fixing cyanobacteria and diatoms, where cyanobacteria fix nitrogen in exchange for amino acids and organic carbon (48). In addition, Amin et al. (18) found that *Sulfitobacter* enhances nitrate uptake and ammonium release in co-cultures, while *Pseudo-nitzschia multiseries* preferentially utilizes ammonium from bacterial sources. This may explain the observed up-regulation of four genes encoding glutamine synthetase in nitrogen metabolism, potentially reflecting microalgae utilization of exogenous ammonium. Carbon (C) and nitrogen (N) are fundamental elements in plant cellular composition, and a higher C:N ratio indicates enhanced nitrogen utilization efficiency (49, 50). Our findings also revealed an elevated C:N ratio in the co-culture, which may be linked to enhanced nitrogen utilization efficiency. It is worth mentioning that due to the limitations of the experiment, we cannot entirely rule out the contribution of *Vibrio* to the total C:N ratio. Therefore, the observed differences may also be attributed to *Vibrio* itself, rather than solely to the hypothesized metabolic changes.

### Chitin metabolism correlates with cell wall defense

A significant alteration in the co-cultured relationship of *T. weissflogii* is the regulation of several genes associated with chitin metabolism, particularly those involved in chitin catabolism (Fig. 4). Chitin is a polymer of β-(1→4)-*N*-acetylglucosamine (GlcNAc), the most abundant polysaccharide in marine environments. It serves a role analogous to cellulose as a principal component of the cell wall in diatoms (Paulsen et al. (51)). The centric diatoms *Thalassiosira* and *Cyclotella* are the primary producers of chitin, with extracellular chitin fibers extending through supporting protrusions in the cell wall (52). As an integral component of the cell wall, chitin provides a site for bacterial attachment in diatoms (16, 53). Chitin biosynthesis and degradation may represent a form of cellular defense. In *T. pseudonana*, abiotic stress upregulates chitin synthase, triggering changes in cell wall morphology (29). Our findings suggest that biotic stress also leads to upregulation of the chitin synthase gene while decreasing chitinase activity, which may collectively enhance chitin biosynthesis and strengthen the cell wall in response to *V. parahaemolyticus*. By contrast, the gene encoding chitinase was upregulated in the transcriptome (Fig. 4). This discrepancy between gene expression and enzyme activity suggests the presence of post-transcriptional regulation of chitinase, warranting further investigation.

### Conclusion

**Fig 6:**
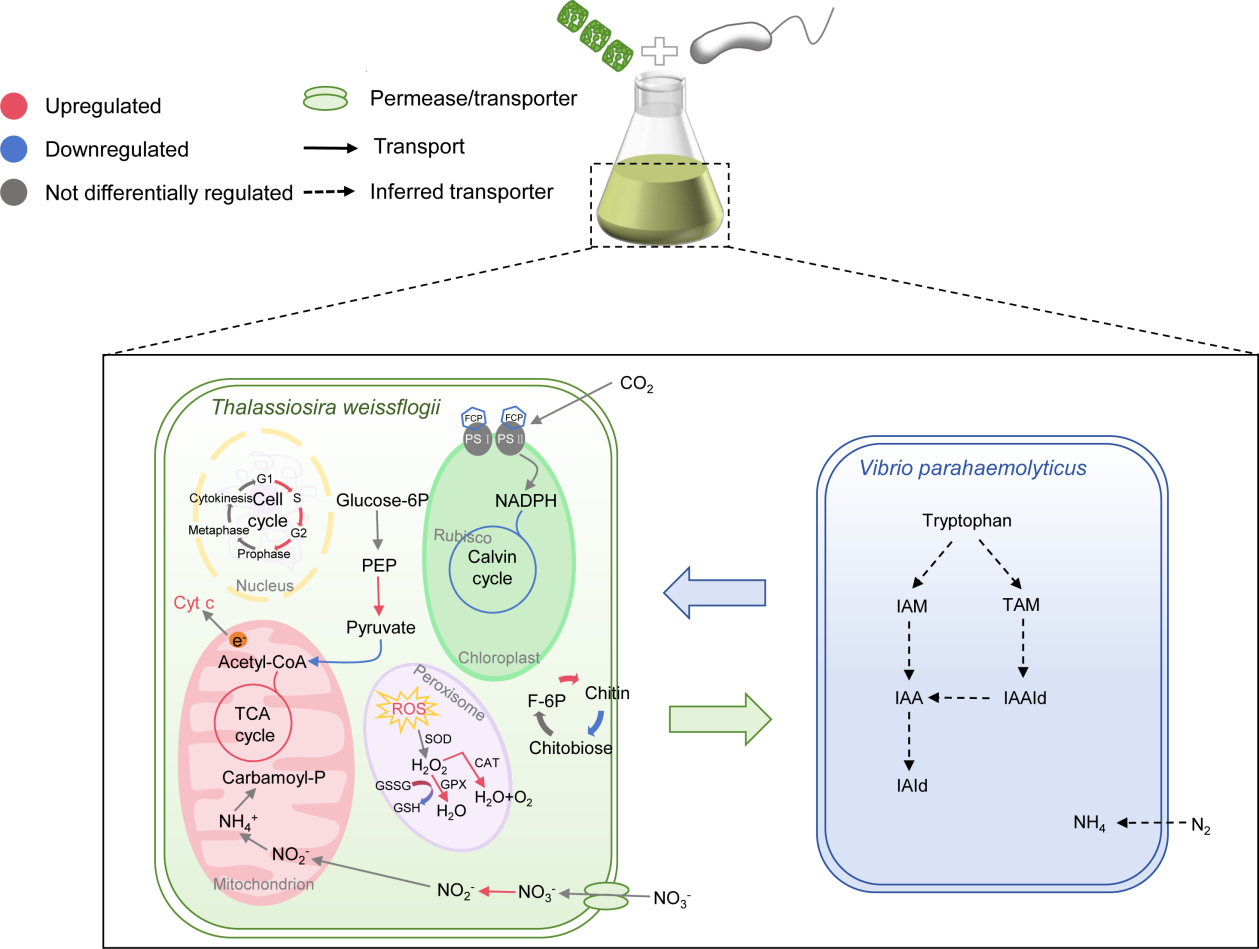
Schematic representation of the proposed model of *T. weissflogii* and *V. parahaemolyticus* interactions, derived from physiological and transcriptomic analyses. Red indicates upregulated genes, blue indicates downregulated genes, and gray represents genes with no differential regulation. Solid arrows denote transport, while dashed arrows represent inferred transport. Different colors are used to distinguish between various organelles.

This study characterizes the physiological and genetic alterations observed in *T. weissflogii* when co-cultured with *Vibrio*. To date, this is the first investigation to explore the molecular mechanisms underlying the effects of *Vibrio* on diatoms. Figure 6 provides a comprehensive overview of the global impact of *V. parahaemolyticus* on the physiology and metabolism of diatoms. Although the PSII of *T. weissflogii* showed no significant changes, chlorophyll biosynthesis and the balance of carbon and nitrogen metabolism were damaged. *T. weissflogii* may sustain regular division and proliferation by upregulating cell cycle genes and the chitin biosynthesis pathway. However, future research should aim to replicate authentic natural environments and utilize techniques such as metagenomics to gain a deeper understanding of diatom-bacteria interactions over extended periods.

## Data Availability

The raw sequence data reported in this paper have been deposited in the National Center for Biotechnology Information (NCBI) under accession no. PRJNA1192336 (transcriptome) and SRR32271522 (reference genome).

## SUPPLEMENTAL MATERIAL

Supplemental figures aem.00323-25-s0001.docx Figures S1 and S2.

Supplemental tables aem.00323-25-s0002.xlsx Tables S1 to S7.

ASM does not own the copyrights to Supplemental Material that may be linked to, or accessed through, an article. The authors have granted ASM a non-exclusive, world-wide license to publish the Supplemental Material files. Please contact the corresponding author directly for reuse.
