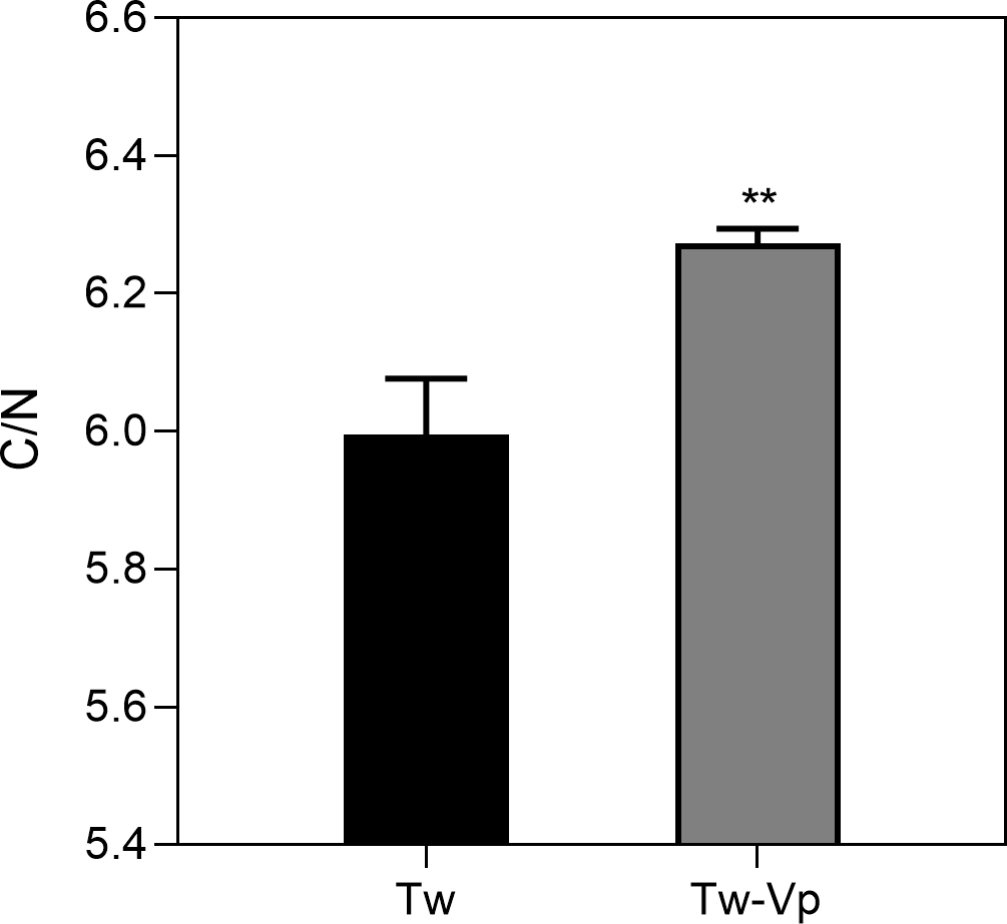# Correction for Wang et al., “Effects of *Vibrio parahaemolyticus* on physiology and metabolism of *Thalassiosira weissflogii* in the co-culture system”

**DOI:** 10.1128/aem.01394-25

**Published:** 2025-08-18

**Authors:** Jiahui Wang, Mengzhen Cheng, Xin Wang, Guangyuan Wang, Delin Duan, Zhanru Shao

## AUTHOR CORRECTION

Volume 91, no. 5, e00323-25, 2025, https://doi.org/10.1128/aem.00323-25. Page 6: Figure 2D should appear as shown in this correction. In the published article, Fig. 2B and D were duplicated.

Page 6, line 14: “A total of 1,536 DEGs” should read “A total of 1,527 DEGs.”

We sincerely apologize for these errors, which did not alter the final results.

**FIG 2:**