# Supplementary material for: Effects of Vibrio parahaemolyticus on physiology and metabolism of Thalassiosira weissflogii in the co-culture system
Source: Appl Environ Microbiol. 2025 Apr 17;91(5):e00323-25. doi: 10.1128/aem.00323-25 (PMC12094009; doi:10.1128/aem.00323-25)
Supplement: Supplemental figures — Figures S1 and S2. [file aem.00323-25-s0001.docx]

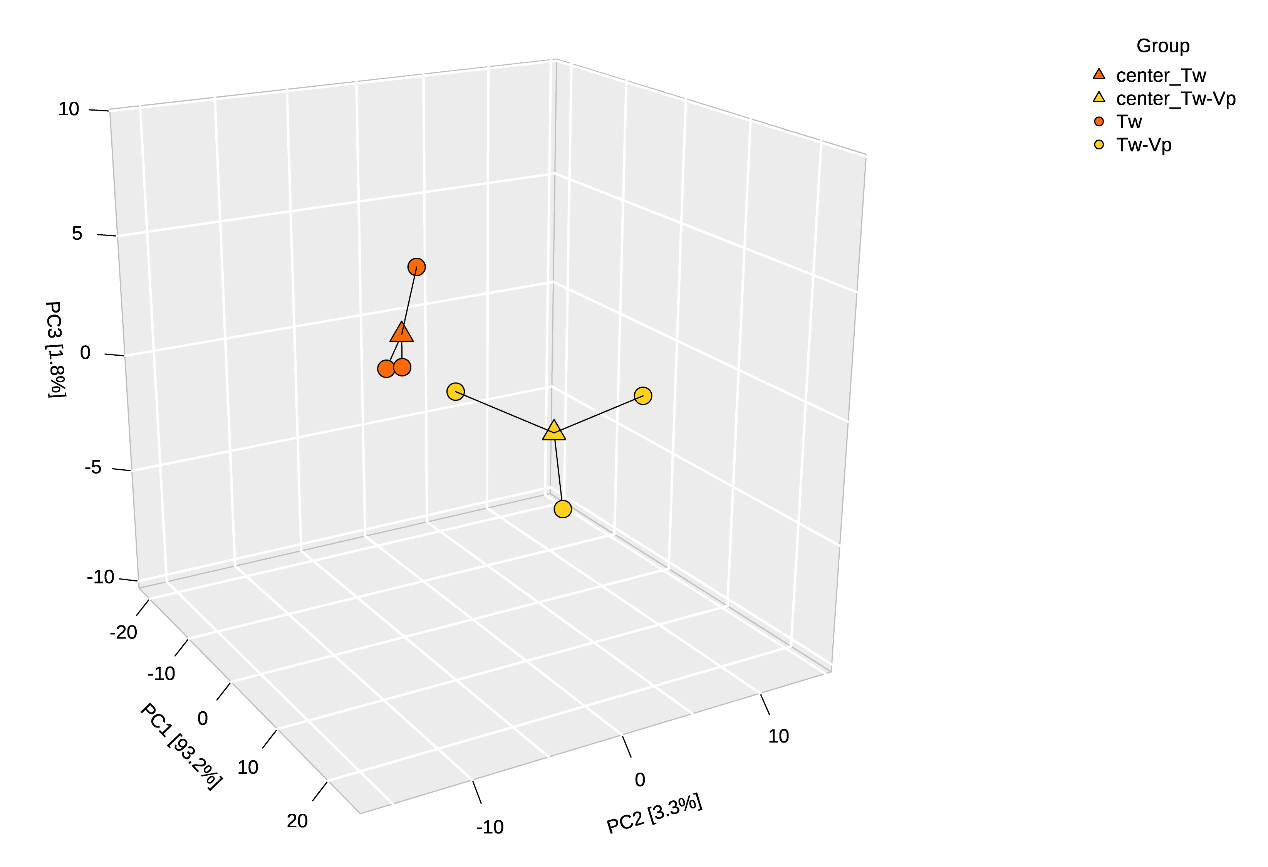


**Fig. S1** Principal component analysis. x-axis is the first principal component, y-axis is the second principal component and z-axis is the third principal component, with different colours indicating different groupings.


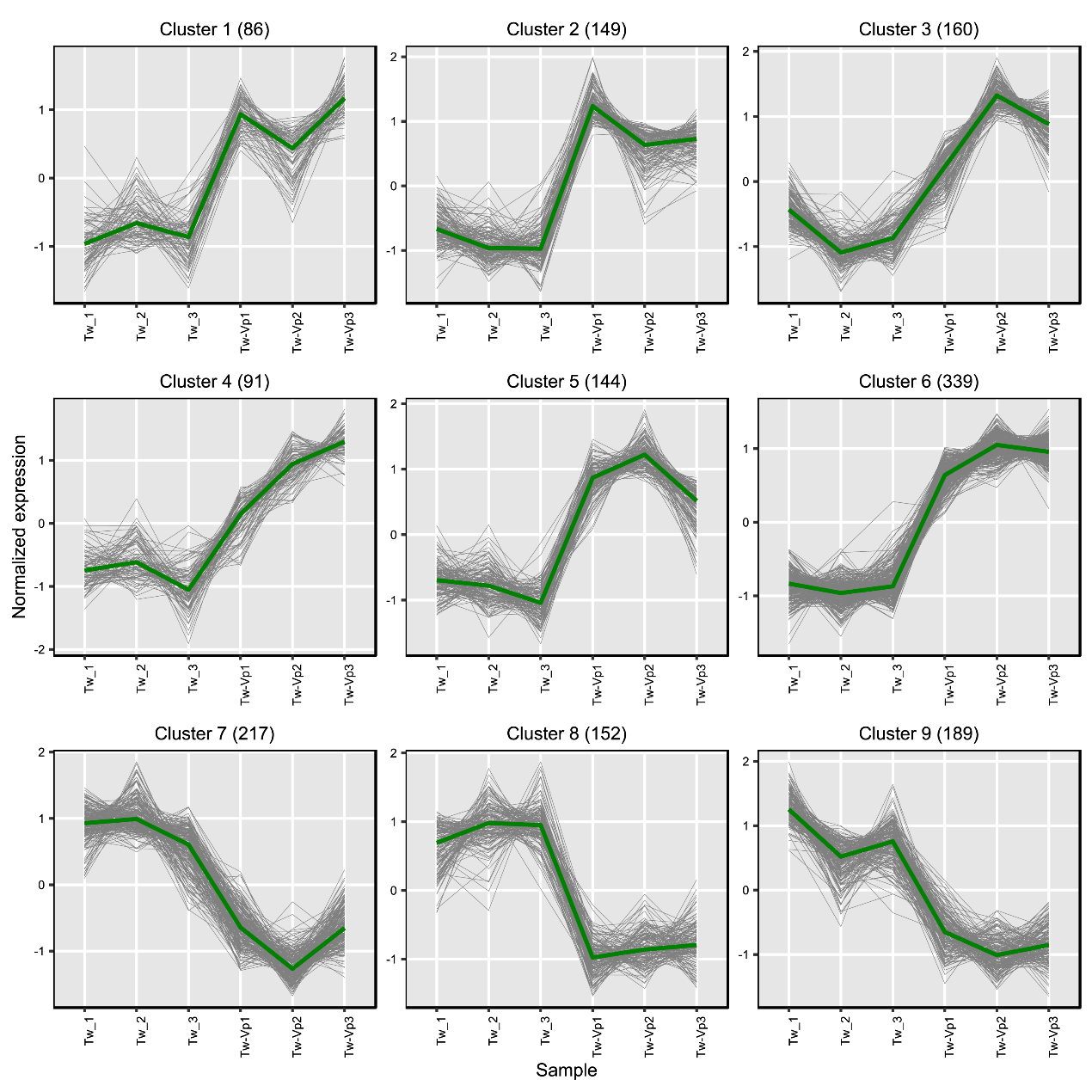


**Fig. S2** Plot of differential gene expression trend analysis. Numbers in parentheses indicate the number of genes in each Cluster, the background line in the graph shows the expression pattern of genes in each Cluster, and the middle line indicates the average of the expression of all genes in the Cluster in the sample.
